# Brodalumab for the Treatment of Moderate-to-Severe Psoriasis: An Expert Delphi Consensus Statement

**DOI:** 10.3390/jcm12103545

**Published:** 2023-05-18

**Authors:** Maria Concetta Fargnoli, Federico Bardazzi, Luca Bianchi, Paolo Dapavo, Gabriella Fabbrocini, Paolo Gisondi, Giuseppe Micali, Anna Maria Offidani, Giovanni Pellacani, Nevena Skroza, Rosa Giuseppa Angileri, Martina Burlando, Anna Campanati, Carlo Giovanni Carrera, Andrea Chiricozzi, Andrea Conti, Clara De Simone, Vito Di Lernia, Enzo Errichetti, Marco Galluzzo, Claudio Guarneri, Claudia Lasagni, Serena Lembo, Francesco Loconsole, Matteo Megna, Maria Letizia Musumeci, Francesca Prignano, Antonio Giovanni Richetta, Emanuele Trovato, Marina Venturini, Ketty Peris, Piergiacomo Calzavara Pinton

**Affiliations:** 1 Department of Biotechnological and Applied Clinical Sciences, University of L’Aquila, 67100 L’Aquila, Italy; 2 Dermatology Unit, Ospedale San Salvatore, 67100 L’Aquila, Italy; 3 Dermatology Unit, IRCCS Azienda Ospedaliero-Universitaria di Bologna, Policlinico S. Orsola Malpighi, 40126 Bologna, Italy; federico.bardazzi@aosp.bo.it; 4 Department of Systems Medicine, University of Rome “Tor Vergata”, 00133 Rome, Italy; bianchiluca264@gmail.com (L.B.); marco.galluzzo83@gmail.com (M.G.); 5 Dermatology Unit, Azienda Ospedaliera Universitaria “Policlinico Tor Vergata”, 00133 Rome, Italy; 6 Dermatology Clinic, Department of Medical Sciences, University of Turin, 10124 Turin, Italy; paolo.dapavo@gmail.com; 7 Section of Dermatology, Department of Clinical, Medicine and Surgery, University of Naples Federico II, 80138 Naples, Italy; gafabbro@unina.it (G.F.); mat24@libero.it (M.M.); 8 Section of Dermatology and Venereology, Department of Medicine, University of Verona, 37129 Verona, Italy; paolo.gisondi@univr.it; 9 Department of Dermatology, University of Catania, 95123 Catania, Italy; gimicali1@hotmail.it (G.M.); musumecimarialetizia@gmail.com (M.L.M.); 10 Department of Clinical and Molecular Sciences, Dermatology Unit, Polytechnic Marche University, 60121 Ancona, Italy; a.offidani@ospedaliriuniti.marche.it; 11 Dermatology Clinic, Department of Clinical Internal, Anesthesiologic and Cardiovascular Sciences, Sapienza Medical School, Sapienza University of Rome, 00185 Rome, Italy; giovanni.pellacani@uniroma1.it; 12 Dermatology Unit “D. Innocenzi”, Department of Medical and Surgical Sciences and Biotechnologies, Sapienza University of Rome-Polo Pontino, 04100 Latina, Italy; nevena.skroza@uniroma1.it; 13 UOC Dermatologia, ARNAS Civico Di Palermo, 90127 Palermo, Italy; rosagiuseppaangileri@yahoo.it; 14 Clinica Dermatologica, DissaL, Ospedale Policlinico San Martino-IRCCS, 16132 Genova, Italy; martinaburlando@hotmail.com; 15 Department of Clinical and Molecular Sciences, Dermatology Clinic, Polytechnic Marche University, 60121 Ancona, Italy; anna.campanati@gmail.com; 16 Dermatology Unit, Department of Internal Medicine, Fondazione IRCCS Ca’ Granda Ospedale Maggiore Policlinico, 20122 Milan, Italy; carlogcarrera@gmail.com; 17 Dermatologia, Dipartimento di Medicina e Chirurgia Traslazionale, Università Cattolica del Sacro Cuore, 00168 Rome, Italy; chiricozziandrea@gmail.com (A.C.); clara.desimone@unicatt.it (C.D.S.); ketty.peris@unicatt.it (K.P.); 18 UOC di Dermatologia, Dipartimento di Scienze Mediche e Chirurgiche, Fondazione Policlinico Universitario A. Gemelli–IRCCS, 00168 Rome, Italy; 19 Dermatologic Unit, Department of Surgery, Infermi Hospital, AUSL Romagna, 47923 Rimini, Italy; a.conti.dermo@gmail.com; 20 Dermatology Unit, Arcispedale S. Maria Nuova, Azienda USL-IRCCS di Reggio Emilia, 42122 Reggio Emilia, Italy; dilernia.vito@ausl.re.it; 21 Institute of Dermatology, Azienda Sanitaria Universitaria Friuli Centrale (ASU FC), University of Udine, 33100 Udine, Italy; enzoerri@yahoo.it; 22 Department of Biomedical and Dental Sciences and Morphofunctional Imaging, University of Messina, 98122 Messina, Italy; cguarneri@unime.it; 23 Clinica Dermatologica, Dipartimento delle Medicine Specialistiche AOU Policlinico di Modena, 41121 Modena, Italy; lasacla65@gmail.com; 24 Department of Medicine, Surgery and Dentistry, “Scuola Medica Salernitana”, University of Salerno, 84084 Fisciano, Italy; slembo@unisa.it; 25 Department of Biomedical Sciences and Human Oncology, University of Bari Aldo Moro, 70121 Bari, Italy; franciscus59@gmail.com; 26 Azienda Ospedaliero Universitaria Consorziale Policlinico di Bari, 70124 Bari, Italy; 27 Department of Health Sciences, Section of Dermatology, University of Florence, 50125 Florence, Italy; francesca.prignano@unifi.it; 28 Unit of Dermatology, Department of Internal Medicine and Medical Specialties, Sapienza University of Rome, 00185 Rome, Italy; antonio.richetta@uniroma1.it; 29 Section of Dermatology, Department of Medical, Surgical and Neurological Science, S. Maria alle Scotte Hospital, University of Siena, 53100 Siena, Italy; emanuele.trovato@ao-siena.toscana.it; 30 Dermatology Department, University of Brescia, 25121 Brescia, Italy; marina.venturini@unibs.it (M.V.); piergiacomo.calzavarapinton@unibs.it (P.C.P.)

**Keywords:** Delphi, consensus, statement, brodalumab, psoriasis

## Abstract

Brodalumab is a recombinant, fully human immunoglobulin IgG2 monoclonal antibody specifically targeted against interleukin-17RA that has been approved for the treatment of moderate-to-severe psoriasis in Europe. We developed a Delphi consensus document focused on brodalumab for the treatment of moderate-to-severe psoriasis. Based on published literature and their clinical experience a steering committee drafted 17 statements covering 7 domains specific to the treatment of moderate-to-severe psoriasis with brodalumab. A panel of 32 Italian dermatologists indicated their level of agreement using a 5-point Likert scale (from 1 = “strongly disagree” to 5 = “strongly agree”) using an online modified Delphi method. After the first round of voting (32 participants), positive consensus was reached for 15/17 (88.2%) of the proposed statements. Following a face-to-face virtual meeting, the steering committee decided that 5 statements would form “main principles” and 10 statements formed the final list. After a second round of voting, consensus was reached in 4/5 (80%) of the main principles and 8/10 (80%) for consensus statements. The final list of 5 main principles and 10 consensus statements identify key indications specific to the use of brodalumab in the treatment of moderate-to-severe psoriasis in Italy. These statements aid dermatologists in the management of patients with moderate-to-severe psoriasis.

## 1. Introduction

Psoriasis is a chronic inflammatory skin disease affecting up to 3% of individuals worldwide, with a considerable impact on patient quality of life (QoL) and healthcare costs [1,2,3], and up to one-third of patients have signs and symptoms of moderate-to-severe disease [4,5].

The clinical presentation of plaque psoriasis can vary considerably in terms of age of onset, signs and symptoms, involved body areas, and disease severity [3].

Treatment regimens should be tailored to the patient to meet needs in relation to disease severity, impact on QoL, response to prior therapies, and the presence of comorbidities [4,5,6].

Moderate-to-severe psoriasis requires the use of systemic treatments (conventional or biological) and several biologics—including tumor necrosis factor α (TNFα) inhibitors (adalimumab, infliximab, etanercept, and certolizumab pegol), the interleukin (IL)-12/IL-23 inhibitor ustekinumab, IL-17A inhibitors (secukinumab and ixekizumab), IL-23 inhibitors (guselkumab, tildrakizumab, and risankizumab), and the IL-17A/F inhibitor bimekizumab—have shown substantial efficacy in lesion clearance and improvement in symptoms of moderate-to-severe plaque psoriasis [7,8,9]. 

Recent Italian guidelines (that are adapted from European guidelines) suggest that certain parameters should be taken into consideration when choosing biological drug treatment, such as disease-, patient- and treatment-related factors [2,10,11,12]. In this regard, it is important in real-life clinical practice to define the optimal biologic for a given patient from a “best treatment” perspective, through an analysis of the characteristics of the drug with regard to the specific needs of the patients and their disease features.

Brodalumab is an anti-IL-17 receptor A antibody that has been approved for the treatment of moderate-to-severe plaque psoriasis in patients who are eligible for biological therapy and those with inadequate response to other systemic therapies [13]. It has a unique mechanism of action compared to other IL-17A agents since it selectively binds to the A subunit of the IL-17 receptor and blocks the biological activity of the pro-inflammatory cytokines IL-17 (IL-17A, IL-17F, IL-17A/F heterodimer, IL -17C, and IL-17E [IL-25]), resulting in anti-inflammatory effects and improvement in clinical symptoms associated with psoriasis [13,14,15,16,17,18,19].

Besides its unique mechanism of action, brodalumab may be considered an effective treatment in patients unresponsive to other biologics that have a different mechanism of action [20], particularly in patients after primary or secondary failure with other anti-IL17As [21,22]. A broader inhibition of IL-17 as in the case of brodalumab, could represent a favorable factor in the choice of a therapy for the treatment of psoriasis [21].

Administration of 210 mg brodalumab every 2 weeks provides a rapid improvement in disease severity and a good safety profile in moderate-to-severe psoriasis [7,23,24,25,26,27]. In addition, data from comparative studies show a faster onset of action with brodalumab compared to other biologic therapies [26,28]. 

In a pooled analysis of data from the two phase 3 trials AMAGINE-2 and AMAGINE-3, brodalumab was shown to have a rapid onset of action and to rapidly improve disease severity [26]. The median time taken for 25% of patients to achieve a PASI 75 response was 2.1 weeks for brodalumab compared to 4.8 weeks for ustekinumab. Furthermore, a recent systematic review by Egeberg examined the time to onset of action for IL-17 and IL-23 agents for the treatment of psoriasis [28]. A total of 27 studies were included and the shortest time to 25% and 50% of patients to achieve PASI 90 were seen with 210 mg brodalumab given every 2 weeks (Q2W; 3.5 weeks and 6.2 weeks, respectively) followed by 80 mg ixekizumab (Q2W (4.1 and 7.4 weeks, respectively) and 80 mg ixekizumab (Q4W 4.6 and 8.1 weeks, respectively). 

A rapid onset of action, as in the case of brodalumab, could be advantageous, particularly, in psoriasis patients with severe disease where their QoL may be compromised [28]. In addition to improved skin clearance (PASI 100 of 37–44% in brodalumab vs. 19–22% for ustekinumab at 12 weeks) [24], brodalumab has also demonstrated dramatic and rapid improvement in symptoms, including pruritus (PSI-Itch of 71.4% for brodalumab vs. 63.6% for ustekinumab at 12 weeks) as well as in the treatment of difficult-to-treat areas such as the scalp [23,24,29] and nails [30]. 

Currently, based on the available literature, there is no consensus document on the use of brodalumab for the treatment of moderate-to-severe psoriasis. Based on this premise, the aim of this expert consensus statement was to evaluate current literature on the use of brodalumab for the treatment of moderate-to-severe psoriasis considering its mechanism of action, efficacy, safety, and use in patients with comorbid diseases, together with expert opinion, to identify the best areas for brodalumab treatment in real-life clinical practice. 

## 2. Methods

### 2.1. Study Aims

The objective of the SPARK study was to develop a Delphi consensus statement focused on the best treatment of moderate-to-severe psoriasis with brodalumab. A panel of dermatologists with expertise in psoriasis reviewed and evaluated the available literature to define the best areas of action of brodalumab with a view to the best treatment, based on the drug’s properties and considering specific patient needs and their disease characteristics. 

### 2.2. Study Design

**Figure 1:**
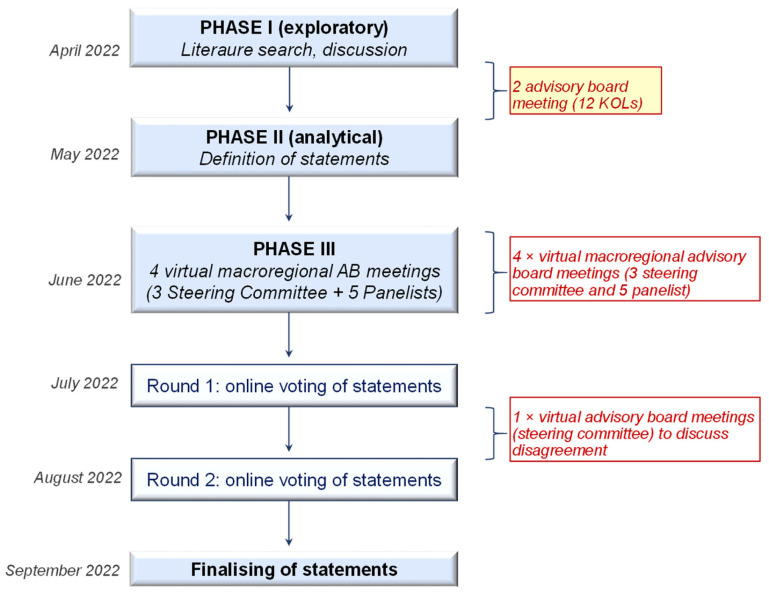
Main stages undertaken in the flow of the Consensus (SPARK) study.

The SPARK study included a modified Delphi process and was organized in the following 3 phases over a period of 6 months (March 2022 to September 2022) (Figure 1).

The project involved the participation of 32 dermatologist key opinion leaders (KOLs): 12 of whom were members of the steering committee and the other 20 KOLs had the role of panelist. The study consisted of three phases:

(a) Phase I (exploratory phase): The steering committee was responsible for the preliminary literature review and subsequent development of statements to be included and the definition of a cut-off value for cumulative agreement. Statements characterizing brodalumab were developed through two virtual advisory board meetings, upstream of which a bibliographic search was carried out on the use of brodalumab in psoriasis, shared with the steering committee as a basis for discussion and eventual revision.
(b) Phase II (analytical phase): Statements were developed by the steering committee.
(c) Phase III: A series of 4 macro-regional virtual meetings were undertaken, each having the participation of 8 KOLs: 3 steering committee members and 5 panelists.

### 2.3. Scientific Board

The scientific board comprised Italian dermatologists with particular interest in psoriasis according to publication records, participation in national/international meetings, clinical trials, expertise in the integrated design and management of different clinical scenarios, and Delphi consensus statements and/or senior academic rank. 

### 2.4. Literature Review

The aim of the literature search was to identify published studies specifically related to the use of brodalumab for the treatment of moderate-to-severe psoriasis. A literature search was performed only on articles published in peer-reviewed journals, to ensure the methodological quality of studies examined and conclusions drawn. A systematic electronic search was performed using the PubMed/Medline database (up to 1 March 2022).

Keywords that were applied to the search field included: (“brodalumab” [Supplementary Concept] OR brodalumab OR KHK-4827 OR KHK4827 OR AMG-827 OR “AMG 827” OR AMG827) AND (“Psoriasis” [Mesh] OR psoriasis* OR psoriatic* OR PsO)” using Boolean operators either alone or in different combinations. 

The search results only considered meta-analyses, systematic reviews, clinical studies, guidelines, or real-world data published in the English, French, Italian, Spanish, and German languages (unless a specific article in another language was considered relevant by the Board). Reviews, letters, and abstracts were excluded from the search.

### 2.5. First and Second Round Online Delphi Voting

Potential statements, specific to issues relating to psoriasis patients and the use of brodalumab based on expert opinion and from literature reviews, were voted on by each member of the expert panel (32 dermatologists) through an online survey (www.surveymonkey.com with Verisign certificate version 3, 128-bit encryption). Voting was undertaken using a 5-point Likert scale to indicate their level of agreement on each statement: (1) absolutely disagree, (2) disagree, (3) neither agree nor disagree, (4) agree, (5) absolutely agree. The results of the data analysis derived from the survey were expressed as percentage response for each item. A total cumulative agreement was defined as the sum of response percentage in items 4 (“agree”) and 5 (“absolutely agree”). For the purpose of this consensus, a total cumulative agreement ≥75% was considered a priori to represent consensus for each statement. This definition of agreement was based on standards used in previous Delphi studies [31,32,33]. During the first round of Delphi voting, which was performed anonymously, every participant had the possibility to consult literature on the topic. 

Responses of participants after the first round of voting were collected and analyzed by the steering committee. All statements without consensus were discussed and modified as required. Participants voted again on all the statements using the same 5-point scale. The results of this second voting were expressed as total cumulative agreement (TCA) percentage for each item. 

## 3. Results

### 3.1. Literature Search

**Figure 2:**
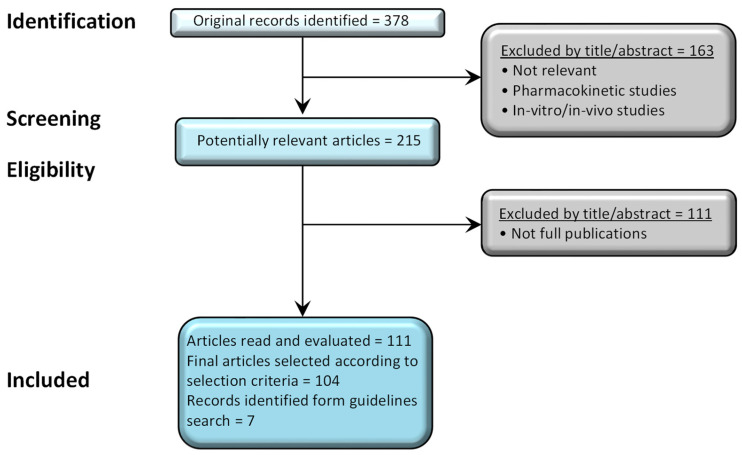
Selection process for studies included in the literature search.

A flow diagram of the selection process for the literature search is shown in Figure 2. The initial search returned a total of 378 distinct results, a total of 163 records were excluded after reading the title, abstract, full text, or publications that were considered irrelevant or not clinical studies. A further 111 studies were excluded as they were not full publications according to our inclusion criteria. The remaining 104 articles were considered potentially relevant for further evaluation. 

### 3.2. Development of Statements and Delphi Voting

Based on the literature search, the steering committee considered 7 key core areas (domains) (Table 1) that were used to develop 17 statements (Supplementary Table S1). After the first round of voting (32 participants), positive consensus was reached for 15/17 (88.2%) of the proposed statements. Following the comments received from the expert panel, the steering committee decided that 5 statements would form the “main principles” (mean TCA = 94.6%, range 90.6–100%) and 10 statements (2 statements were modified/merged from 4 from the original 17) (Supplementary Table S2) to form the final list (Table 2) of which 5 statements (only statements that were modified) were voted on in a second round with consensus reached in 4/5 (80%). For one statement that was further revised, a consensus was not reached (65.6%) and a second statement was not further voted on. After the second round of voting, a positive consensus was reached for 8/10 (80%) statements, with a mean final TCA of 87.2% (ranging between 59.4–96.9%) (Table 2). The overall response rate was 100% for the first and second rounds of voting.

## 4. Discussion

This study presents results obtained by the combination of a Delphi survey involving 32 Italian dermatologists following face-to-face virtual meetings and two rounds of Delphi voting with the aim to identify and define statements on the appropriate treatment of moderate-to-severe psoriasis with brodalumab. The development of the main principles and final statements were based on the principles of evidence-based medicine supported by a systematic literature review and an iterative anonymous voting process.

### 4.1. Main Principles (Statements 1–5)

After the first round of voting, the panel decided that five statements (of the original 17 that were proposed) could be transformed into stand-alone statements/principles that had achieved a collective high level of agreement among the panel (mean TCA = 94.6%, range 90.6–100%). The five statements (Table 2) were focused on the therapeutic indication, impact on QoL, and safety profile and were considered as concepts agreed upon by all participants where there was no doubt and also on the basis of therapeutic indication of brodalumab in psoriasis according to European (EMA), American (FDA) regulatory bodies and the Italian Medicines Agencies (AIFA) [13,34,35]. 

It was the opinion of the Expert Panel that in patients who are not responsive to conventional treatment such as cyclosporine, the disease may be difficult to control upon discontinuation of cyclosporine therapy, requiring the use of a fast-acting drug. Therefore, having a fast-acting drug like brodalumab that can quickly control the disease may be advantageous. In cases of inflammatory psoriasis initially treated using cyclosporine, brodalumab is particularly suitable for this type of psoriasis when it is necessary to switch from a traditional drug such as cyclosporine to a biologic within a few months.

In addition, the use of brodalumab may be considered in patients where TNF-alpha is contraindicated or not responsive to TNF-alpha or not appropriate, such as those patients with multiple sclerosis [36,37] or congestive heart failure [38,39]. In these patients where TNF-alpha is contraindicated, it is also important to consider the type of contraindication that patients have to TNF-alpha (e.g., active tuberculosis is also a contraindication for brodalumab). 

Numerous trials and real-life studies have shown that the treatment with brodalumab is associated with improvement in disease severity, signs and symptoms, and high rates of skin clearance that is closely correlated to an improvement in QoL [40,41,42,43].

Candida infection may appear with anti-IL-17 treatment, given the role of interleukin 17 in protection against bacterial and fungal infections. Frequently, this infection can easily be managed and treated by a dermatologist [44,45]. Relatively low rates of Candida infection have been observed with long-term brodalumab treatment [46]. Overall, however, the rates of, and discontinuations due to, candidiasis are extremely low in patients treated with anti-IL-17 inhibitors. Patients are, therefore, able to continue therapy without interruption of treatment [47]. The safety of brodalumab has been evaluated in clinical trials and recent data are also available from a recent 4 year pharmacovigilance report by Lebwohl and colleagues based on data from 4,019 patients (estimated brodalumab exposure of 4,563 patient-years) [27]. The most common adverse event was arthralgia (115 events; 2.52 events per 100 patient-years). No suicides were reported. There were 102 cases with serious infections that did not include serious fungal infections. There were no new cases of Crohn’s disease. These 4-year pharmacovigilance data corroborate with data from long-term clinical trials and 3-year pharmacovigilance data.

From the experience of real-life clinical practice of the board, no cases of first diagnosis of chronic inflammatory bowel disease have been observed with brodalumab (non-differentiating aspect compared to other IL-17A inhibitors). Like other anti-IL-17 drugs, brodalumab is not indicated in psoriatic patients with active IBD [41]. 

### 4.2. Consensus Statements (No. 1–10)

Statements were commented on in further detail by the Board and these are outlined in detail below:

**Comments specific to statement No. 1:** “*Brodalumab, with its unique mechanism of action, represents an appropriate therapeutic option in psoriatic patients non-responsive to anti-IL-17A agents*”.

The Expert Panel highlighted that given the unique mechanism of action of brodalumab [48], the intraclass switch can be justified to a greater extent [22,49,50]. In a multicenter retrospective study by Yeung et al., 47 patients with chronic plaque psoriasis were treated with brodalumab after discontinuation of secukinumab or ixekizumab due to nonresponse [49]. Of the 47 patients, 20 (42.5%) achieved PASI 100 with brodalumab at week 16 and PASI 90 and PASI 75 at week 16 were achieved by 22 (46.8%) and 29 (61.7%) patients, respectively. In an open-label multicenter study conducted on 39 patients with moderate-to-severe psoriasis, Kimmel and colleagues evaluated the use of brodalumab in patients who had experienced previous failure to secukinumab or ixekizumab [22]. PASI 75, PASI 90, and PASI 100 response was achieved in 76%, 50%, and 32% of patients, respectively, and 71% of patients achieved an sPGA of 0 or 1 [22]. In another study by Loft and colleagues, patients experiencing previous failure to an IL-17 blocker were switched to brodalumab and 70% of patients achieved a PASI 75 response after 12 weeks [50]. The Panel also noted that it is also worth considering patients that have experienced paradoxical/eczematous reactions from the use of other IL-17A inhibitors; these patients may, therefore, be candidates for switching to brodalumab [51].

**Comments specific to statement No. 2:** “*It is the opinion of the Expert Panel that brodalumab may also be effective in psoriatic patients non-responsive to IL-23 inhibitors*”.

Besides being effective in patients who are not responsive to conventional treatment (Statement No. 2; Main Principles) or not responsive to anti-TNF alpha inhibitors (Statement No. 3; Main Principles), a high level of agreement was reached by the panel on the use of brodalumab in patients non-responsive to other biologics such as IL-23 inhibitors [52]. Despite the limited evidence available from RCTs, this statement still achieved a collective agreement from the Panel that was based on their clinical experience.

**Comments specific to statement No. 3:** “*The speed of action of brodalumab makes it an appropriate choice in patients where a rapid therapeutic response is necessary*”.

Brodalumab is characterized by its rapid action in patients with moderate-to-severe psoriasis as documented in pooled analysis of the two phase-3 trials AMAGINE-2 and AMAGINE-3 trials compared to ustekinumab as well as a systemic review of 27 studies [26,28]. 

The multifaceted burden (e.g., pain, itching, psychosocial, and QoL) experienced in patients living with moderate-to-severe psoriasis [1], further necessitates the availability of a treatment approach that is rapid and effective. As previously alluded to (Main Principles), the Expert panel also highlighted that the speed of action of brodalumab is particularly effective in patients at risk of relapse/rebound psoriasis after systemic withdrawal, as in the case of cyclosporine and in patients where it is crucial to act quickly because their QoL is compromised.

**Comments specific to statement No. 4:** “*Brodalumab, due to its rapid action on itching, represents an appropriate therapeutic choice in patients where itching affects their quality of life*”.

Pruritus or itching is the most commonly reported symptom in patients with psoriasis and this symptom is correlated with QoL impairment, stigmatization, and depressive symptoms [53] and, in severe cases, leads to sleep deprivation [54]. In psoriasis patients with pruritus, brodalumab has been shown to be rapid and effective for the treatment of pruritus [29,55], therefore, justifying the statement from the Panel that it would represent an appropriate therapeutic choice in these patients.

**Comments specific to statement No. 5:** “*Brodalumab represents an appropriate therapeutic choice when there is involvement of difficult-to-treat areas, such as scalp/nails or palmo-plantar area*.”

Approximately two-thirds of patients have psoriatic lesions in difficult-to-treat areas typically involving sites such as the scalp, nails, genital area, and the palmo-plantar area [6,56,57,58].

Scalp involvement is estimated to occur in nearly 80% of individuals with psoriasis [59] and is associated with negative effects on QoL, including intense itch, embarrassment due to scale shedding, and distress over physical appearance [60]. Approximately half of all patients with psoriasis have nail involvement [61,62] which can also be painful and impact their QoL. Furthermore, as many as one-third of patients with psoriasis are also affected by psoriatic lesions in the genital area at some point over the course of their disease [63]. 

There is accumulating evidence from recent studies on the use of brodalumab in difficult-to-treat areas [30,55,64,65]. In a post hoc analysis of pooled data from AMAGINE-2 and AMAGINE-3, significantly more patients treated with brodalumab 210 mg achieved complete clearance (PASI 100) in all body regions after week 52 compared to ustekinumab [66]. In a separate sub-group analysis of the three AMAGINE studies, significant improvements in nail psoriasis with 210 mg and 140 mg brodalumab were observed when compared to placebo at 12 weeks (46.3% and 37.5% vs. 11.6%, respectively; *p* < 0.001) [67]. Scalp psoriasis also showed a rapid response to 12 weeks of treatment with brodalumab [30,68]. The Board highlighted the benefits of treating psoriasis in difficult-to-treat areas particularly seen with regard to itching and improvement in scalp lesions from the first injection with brodalumab. In addition, the Panel noted that the scalp tends to be the first site where there is a clear response to brodalumab as well as the first site where the disease recurs in cases when the therapy fails.

**Comments specific to statement No. 6:** “*Experience from clinical practice suggests that brodalumab may also be effective in erythrodermic psoriatic patients*.”

Although cyclosporine is often used to treat erythrodermal forms of psoriasis, the use of brodalumab after cyclosporine could guarantee effectiveness even in this type of psoriasis [69]. Indeed, evidence from a real-life observational study in Italy [55] and an open-label, multicentre, long-term phase III study in Japanese patients [70] have both shown that brodalumab is effective as a treatment for the erythrodermal forms of psoriasis.

To date, the clinical practice experience of brodalumab for the treatment of erythrodermal psoriasis is limited and it is necessary to objectively rely on the literature. While there is little evidence in the literature, mainly from real-life data and case reports [55,69,70,71], some of the experts involved in the board have had successful experience in clinical practice on the use of brodalumab in erythrodermic patients. In this rare form of psoriasis, this drug, which is characterized by rapid action is strictly needed in favor of other anti-IL17 biologics.

**Comments specific to statement No. 7:** “*Experience from clinical practice indicates that brodalumab may also be effective in pustular psoriasis*.”

The expert panel underlined that the efficacy of brodalumab also depends on the percentage of the pustular component (whether predominant or not): in the palmo-plantar forms with a limited pustular component, the clinical experience is favorable, whereas, in the forms in which the pustular component is predominant (i.e., exceeds 50%), there is an insufficient clinical experience to validate brodalumab therapy as being effective [55,70,72].

The lack of agreement reached by the panel in the second round of voting reflects differences in experience managing patients with pustular psoriasis among centers. It is agreed among the panel that there is not enough data available in this specific area, mainly limited to several case reports and case series. Limited data on patient-reported instruments are available, and some recent studies are testing new assessment measures [73].

**Comments specific to statement No. 8:** “*Brodalumab exhibits high rates of efficacy and acts with the same rapidity of action in patients undergoing retreatment after its discontinuation*.”

This statement is supported by data from the AMAGINE-1 trial. In a subgroup analysis of the AMAGINE-1 trial [74], patients who achieved PASI 75, PASI 90, and PASI 100 prior to brodalumab withdrawal 100%, 97%, and 95%, respectively, achieved their previous scores after just 24 weeks of retreatment with brodalumab. Furthermore, the improvement in QoL was maintained after retreatment with brodalumab, regardless of exposure to prior biologic treatment. In the long-term subgroup analysis of the AMAGINE-1 trial [40], following withdrawal from brodalumab, the return of disease occurred after 74.7 ± 50.5 days. Overall, 88% of patients achieved sPGA 0/1 at week 120 and 94% achieved PASI 75.

The Expert Panel underline that in their experience the efficacy of brodalumab has been observed to be rapid and maintained following discontinuation of therapy and also for longer periods.

**Comments specific to statement No. 9:** ”*Brodalumab represents an appropriate therapeutic option in obese psoriatic patients and/or in psoriatic patients with metabolic syndrome*.”

Based on their clinical experience and the available documented evidence, the Panel indicates that brodalumab represents an appropriate therapeutic option regardless of the body weight and presence of metabolic syndrome in the psoriatic patient [55,75]. In a real-life retrospective study by Galluzzo et al. in Italy [55], 90 patients with moderate-to-severe psoriasis were treated with 210 mg brodalumab and followed for 1 year. Almost 70% of patients were overweight or obese but both univariate and multivariate analysis did not reveal a significant association between PASI response and body weight or BMI. In the real-life clinical setting, there is evidence that loss of efficacy or primary ineffectiveness may occur for different biological drugs in overweight/obese patients [76,77,78], therefore, justifying the choice of a biologic such as brodalumab that does not lose efficacy in patients with excessive adipose tissue. Indeed, frequently the presence of obesity and metabolic syndrome co-exist therefore brodalumab represents an appropriate therapeutic option in these patients [79]. In addition, the Panel noted that fewer injections are needed compared to other biologics of the same class in obese patients, therefore reducing costs.

**Comments specific to statement No. 10:** “*The presence of non-dominant arthropathy does not preclude the use of brodalumab*.”

Results from the two phase-3 trials AMVISION-1 and -2 demonstrated that brodalumab was associated with a rapid and significant improvement in signs and symptoms of PsA vs. placebo, as well as showing a good tolerability profile [80]. Furthermore, results of phase 3 AMAGINE-2 and -3 trials showed that in patients with concomitant PsA, brodalumab led to rapid and high levels of complete and sustained skin clearance with greater treatment benefit in moderate-to-severe psoriasis vs. ustekinumab [43]. It was the opinion of the expert panel that if the patient has a coexistence of skin and joint involvement and has a confirmed diagnosis of psoriatic arthritis in remission for a long period of time, it is possible to consider using brodalumab in a case-by-case scenario. 

Within the class of IL-17 inhibitors, brodalumab currently has no indication for psoriatic arthritis (PsA). However, brodalumab can be considered in patients with psoriasis and non-dominant arthropathic symptoms given the presence of data supporting the efficacy of brodalumab on the “arthritis” component [80]. The expert panel also concluded that IL-17 is clearly involved in PsA.

## 5. Study Limitations

Some weaknesses of this study need to be addressed. First, the lack of available clinical and scientific data on brodalumab compared to other biologics for most of the issues raised in this Delphi consensus was an important limitation of this study. Second, recent data may have been published subsequent to the literature search, which was performed in the early half of 2022. Third, given the nature of the Delphi method, it is possible that the discussion that preceded the second voting round may have influenced the expert opinion leading to higher percentages of final agreement. Fourth, a Delphi consensus is not a method for introducing new or better evidence, it only proposes a process to identify rational choices on topics not fully supported by solid clinical data. Last, although only 2 of the 10 final statements did not reach an agreement (<75% of the TCA), post-round meetings were informative and helpful in correcting and modifying statements so that they can provide essential guidance for dermatologists in the management of these patients.

## 6. Conclusions

Findings that have emerged from this Delphi consensus statement highlight the best areas of treatment of moderate-to-severe psoriasis with brodalumab. The expert panel drafted 5 main principles and 10 consensus statements. These statements should provide dermatologists with a summary of core issues to aid in the management of patients. 

A high proportion of patients with moderate-to-severe psoriasis are burdened with comorbid diseases. Given the added benefit of brodalumab in these patients and in an effort to improve the therapeutic management of these patients, future consensus or shared discussion between dermatologists and specialists in other areas such as diabetes and rheumatology is warranted. 

## Figures and Tables

**Table 1:** Core domains proposed by the panel to develop statements on the use of brodalumab as appropriate treatment for moderate-to-severe psoriasis.

No.	Domain and Description
1	*Mechanism of action/effectiveness/safety in patients with previous failure/multifailure.* Literature suggests that brodalumab may represent a good therapeutic option both in patients who are bionaïve and in patients not responsive to other biological drugs, even after primary failure, including anti IL-17A agents.
2	*Effectiveness/rapidity of action and severity of disease.* A rapid onset of action, as in the case of brodalumab, could be advantageous, particularly in psoriasis patients with severe disease.
3	*Efficacy at different sites.* Literature on the use of brodalumab on scalp and in nail psoriasis may be considered by clinicians when choosing this treatment.
4	*Long-term effectiveness/recapture rate/immunogenicity patient discontinuation, relapse.* Literature shows that brodalumab is an effective long-term treatment option for psoriasis even after suspension. Brodalumab shows a low incidence of immunogenicity and high rates of regaining efficacy after withdrawal.
5	*Mechanism of action/effectiveness/safety in patients with comorbidities.* Literature shows the multiple uses of brodalumab in a broad spectrum of patients with comorbidities such as obesity, hypertension, metabolic syndrome, and psoriatic arthritis.
6	*Impact on quality of life.* Treatment with brodalumab shows a positive impact on the quality of life of patients with psoriasis, both in bionaïve patients and in patients not responsive to other biological drugs, with significant improvement also with respect to aspects related to work-related issues (absenteeism, work impairment, and productivity).
7	*Safety.* Brodalumab has a favorable safety profile; it is safe and well tolerated both in the early and long-term, with relatively low rates of adverse events of particular concern, such as candida infection.

**Table 2:** Final list of Main Principles (N = 5) and Consensus Statements (N = 10).

**No.**	**Main Principles**	
1	Brodalumab is indicated for the treatment of moderate-to-severe plaque psoriasis in adult patients who are candidates for systemic therapy.	Not voted on
2	Brodalumab represents an appropriate therapeutic choice in bionaïve psoriatic patients either non-responsive to conventional drugs or in whom conventional drugs are contraindicated.	Not voted on
3	Brodalumab represents an appropriate therapeutic option in psoriatic patients non-responsive to TNF-alpha inhibitors or in bionaïve psoriatic patients who have contraindications to TNF-alpha inhibitors.	Not voted on
4	Brodalumab shows a positive impact on the quality of life of psoriatic patients, both bionaïve and non-responsive to conventional treatments or other biological drugs, with significant improvements in work productivity.	Not voted on
5	Brodalumab has a favorable safety profile with few and easily manageable adverse events, such as Candida infections which do not represent a relevant clinical problem.	Not voted on
		
**No.**	**Revised statements after Delphi voting**	**Final TCA (%)**
1	Brodalumab, with its unique mechanism of action, represents an appropriate therapeutic option in psoriatic patients non-responsive to anti-IL-17A agents.	93.8
2	It is the opinion of the Expert Panel that brodalumab may also be effective in psoriatic patients non-responsive to IL-23 inhibitors.	93.8
3	The speed of action of brodalumab makes it an appropriate choice in patients where a rapid therapeutic response is necessary.	96.9
4	Brodalumab, due to its rapid action on itching, represents an appropriate therapeutic choice in patients where itching affects their quality of life.	96.9
5	Brodalumab represents an appropriate therapeutic choice when there is involvement of difficult-to-treat areas, such as scalp/nails or palmo-plantar area.	96.9
6	Experience from clinical practice suggests that brodalumab may also be effective in erythrodermic psoriatic patients.	81.3
7	Experience from clinical practice indicates that brodalumab may also be effective in pustular psoriasis.	**65.6**
8	Brodalumab exhibits high rates of efficacy and acts with the same rapidity of action in patients undergoing retreatment after its discontinuation.	93.8
9	Brodalumab represents an appropriate therapeutic option in obese psoriatic patients and/or in psoriatic patients with metabolic syndrome.	93.8
10	The presence of non-dominant arthropathy does not preclude the use of brodalumab.	**59.4**

IL-17 = interleukin-17, TCA = total cumulative agreement. TCA statements not achieving agreement (<75% TCA) are denoted in bold text.

## Data Availability

Not applicable.

## Supplementary Materials

The following supporting information can be downloaded at: https://www.mdpi.com/article/10.3390/jcm12103545/s1, Supplementary Table S1: Results from first round voting of the original 17 proposed statements, Supplementary Table S2: Final list of 10 statements and revision of these statements after Round 1 of Delphi voting.
