# Supplementary material for: Brodalumab for the Treatment of Moderate-to-Severe Psoriasis: An Expert Delphi Consensus Statement
Source: J Clin Med. 2023 May 18;12(10):3545. doi: 10.3390/jcm12103545 (PMC10219103; doi:10.3390/jcm12103545)
Supplement: Supplementary file 1 [file jcm-12-03545-s001.zip › jcm-2323864-supplementary.pdf]

Supplementary Table S1. Results from first round voting of the original 17 proposed statements.

No	Statement	R1 TCA (%)	R2 TCA (%)
1	Brodalumab represents an appropriate therapeutic choice in patients with moderate-to-severe psoriasis.	93.8	
2	Brodalumab represents an appropriate therapeutic option in bio naïve patients with moderate-to-severe psoriasis.	90.6	
3	Brodalumab represents an appropriate therapeutic choice in non-responsive bio naïve psoriatic patients or in whom traditional therapy is contraindicated.	90.6	
4	Brodalumab represents an appropriate therapeutic option in bio naïve patients who have contraindications to anti-TNF-alpha drugs.	90.6	
5	Brodalumab represents an appropriate therapeutic option in psoriatic patients non-responsive to anti-TNF-alpha agents.	96.9	
6	Brodalumab, with its particular mechanism of action, represents an appropriate therapeutic option in psoriatic patients non-responsive to other anti-IL-17A agents.	93.8	
7	It is the opinion of the Expert Panel that brodalumab may also be effective in psoriatic patients resistant (non-responsive) to IL-23 inhibitors.	96.9	93.8
8	The speed of action of brodalumab makes it an appropriate choice in patients where a rapid therapeutic response is necessary.	96.9	
9	Brodalumab, due to its rapid action on itching, represents an appropriate therapeutic choice in patients where itching affects their quality of life.	96.9	
10	Brodalumab represents an appropriate therapeutic choice in the presence of involvement of difficult-to-treat sites, such as scalp/nails.	96.9	96.9
11	Experience from clinical practice suggests that brodalumab may also be effective in erythrodermal psoriatic patients.	81.3	
12	Experience from clinical practice indicates that brodalumab may also be effective in palmar-plantar pustular psoriasis.	**71.9**	**65.6**
13	Brodalumab exhibits high rates of efficacy and acts with the same rapid action in patients undergoing retreatment after discontinuation.	78.1	93.8
14	Brodalumab represents an appropriate therapeutic choice in obese patients and/or patients with metabolic syndrome.	93.8	93.8
15	The presence of non-dominant arthropathic symptoms does not preclude the use of brodalumab.	**59.4**	
16	Brodalumab shows a positive impact on the quality of life of psoriatic patients, both bio naïve and non-responsive to other biological drugs, with significant improvements seen in issues also related to the workplace.	100	
17	Brodalumab has a favourable safety profile in that it is safe and well tolerated, with relatively low rates of adverse events, such as Candida infection which is not a significant clinical problem.	100	

Supplementary Table S2. Final list of 10 statements and revision of these statements after Round 1 of Delphi voting

No.	Revised statements after Round 1 Delphi voting
1	*Brodalumab, with its particular mechanism of action, represents an appropriate therapeutic option in psoriatic patients non-responsive to other anti-IL-17A agents.*<br>***Revised to***<br>Brodalumab, with its unique mechanism of action, represents an appropriate therapeutic option in psoriatic patients non-responsive to ~~*other*~~ anti-IL-17A agents.
2	*It is the opinion of the Expert Panel that brodalumab may also be effective in psoriatic patients resistant (non-responsive) to IL-23 inhibitors.*<br>***Revised to***<br>It is the opinion of the Expert Panel that brodalumab may also be effective in psoriatic patients ~~*resistant*~~ non-responsive to IL-23 inhibitors.
3	The speed of action of brodalumab makes it an appropriate choice in patients where a rapid therapeutic response is necessary.<br>***Not revised***
4	Brodalumab, due to its rapid action on itching, represents an appropriate therapeutic choice in patients where itching affects their quality of life.<br>***Not revised***
5	*Brodalumab represents an appropriate therapeutic choice in the presence of involvement of difficult-to-treat sites, such as scalp/nails.*<br>***Revised to***<br>Brodalumab represents an appropriate therapeutic choice when there is involvement of difficult-to-treat areas, such as scalp/nails or palmo-plantar area.
6	Experience from clinical practice suggests that brodalumab may also be effective in erythrodermic psoriatic patients.<br>***Not revised***
7	*Experience from clinical practice indicates that brodalumab may also be effective in palmar-plantar pustular psoriasis.*<br>***Revised to***<br>Experience from clinical practice indicates that brodalumab may also be effective in ~~palmar-plantar~~ pustular psoriasis.
8	*Brodalumab exhibits high rates of efficacy and acts with the same rapid action in patients undergoing retreatment after discontinuation.*<br>***Revised to***<br>Brodalumab exhibits high rates of efficacy and acts with the same rapidity of action in patients undergoing retreatment after its discontinuation.
9	*Brodalumab represents an appropriate therapeutic choice in obese patients and/or patients with metabolic syndrome.*<br>***Revised to***<br>Brodalumab represents an appropriate therapeutic option in obese psoriatic patients and/or in psoriatic patients with metabolic syndrome.
10	The presence of non-dominant arthropathy does not preclude the use of brodalumab.<br>***Not revised***
